# Analysis of cell-biomaterial interaction through cellular bridge formation in the interface between hGMSCs and CaP bioceramics

**DOI:** 10.1038/s41598-020-73428-y

**Published:** 2020-10-05

**Authors:** Isabel Benjumeda Wijnhoven, Raúl Vallejos, Juan F. Santibanez, Carola Millán, Juan F. Vivanco

**Affiliations:** 1 Facultad de Ingeniería y Ciencias, Universidad Adolfo Ibáñez, Viña del Mar, Chile; 2 Facultad de Artes Liberales, Universidad Adolfo Ibáñez, Viña del Mar, Chile; 3 Group for Molecular Oncology, Institute for Medical Research, University of Belgrade, Belgrade, Serbia

**Keywords:** Biomedical engineering, Biomaterials

## Abstract

The combination of biomaterials and stem cells for clinical applications constitute a great challenge in bone tissue engineering. Hence, cellular networks derived from cells-biomaterials crosstalk have a profound influence on cell behaviour and communication, preceding proliferation and differentiation. The purpose of this study was to investigate in vitro cellular networks derived from human gingival mesenchymal stem cells (hGMSCs) and calcium phosphate (CaP) bioceramic interaction. Biological performance of CaP bioceramic and hGMSCs interaction was evaluated through cell adhesion and distribution, cellular proliferation, and potential osteogenic differentiation, at three different times: 5 h, 1 week and 4 weeks. Results confirmed that hGMSCs met the required MSCs criteria while displaying osteogenic differentiaton capacities. We found a significant increase of cellular numbers and proliferation levels. Also, protein and mRNA OPN expression were upregulated in cells cultured with CaP bioceramic by day 21, suggesting an osteoinductible effect of the CaP bioceramic on hGMSCs. Remarkably, CaP bioceramic aggregations were obtained through hGMSCs bridges, suggesting the in vitro potential of macrostructures formation. We conclude that hGMSCs and CaP bioceramics with micro and macropores support hGMSC adhesion, proliferation and osteogenic differentiation. Our results suggest that investigations focused on the interface cells-biomaterials are essential for bone tissue regenerative therapies.

## Introduction

Ageing is rapidly increasing and will soon become one of the major problems worldwide, with the expected amount of elderly being doubled in the coming 20 years^1^. This will lead to a massive increase in patients presenting bone injuries and deficiencies^2,3^ and thus restoring therapies constitute a great challenge and possess the need to develop new strategies that help treating bone deficits^4^. In the last decades, broad advances in the bone tissue regeneration field using biomaterials such as calcium phosphate based, CaP bioceramics^5,6^. CaP bioceramics, natural and synthetic, have been widely used for medical applications as replacement grafts for the muskulo-skeletal system due to the physical similarity with bone mineral component^7,8^. Natural calcium phosphates occur in the body through either normal or pathological biomineralization, whereas synthetic ones are usually prepared in-vitro by solution-based chemical reactions and a sintering process which affects their mechanical properties^9,10^. Among CaP bioceramics, hydroxyapatite (HA, Ca_10_(PO_4_)_6_(OH)_2_) and tricalcium phosphate (TCP, Ca_3_(PO_4_)_2_) are the most commonly used in clinical applications for bone tissue engineering and are frequently used in the orthopaedic and dental community due to their high biocompatibility and osteoconductivity^3^. Once implanted, bioceramics not only must have the ability to be colonized by host osteo-progenitor cells and blood vasculature but also to induce the capacity of host cells to differentiate to bone cells^11–13^. Another desirable and required property of bioceramics is their bioreabsorbability which can be achieved by cell-mediated processes that avoid potential toxicity of degradation products, allowing the replacement and integration of bioceramics with new natural bone tissue^14,15^. The process of bone regeneration integrated with bioceramics consists on different steps that can be summarized as a complex and multistage process resulting from a plethora of biological stages; including osteogenesis, angiogenesis and inflammatory responses. All these processes are essential for the recovery of tissue homeostasis and function^13^ but for them to occur a good biocompatilibity between the bioceramic and the nearby tissue is essential^12,16^.

In order to improve tissue growth and regeneration, mesenchymal stem cells (MSCs), have been recently included in bone regeneration strategies using CaP bioceramics^6,17^. Different studies have investigated the interaction of MSCs with CaPs and with PLA-CAP^18–20^.

These MSCs constitute a multipotent population that can be harvested from different sources, such as bone marrow, adipose and gingival tissue, among others. MSCs are also a promising source for tissue regeneration, mostly due to their ability to differentiate into various tissues such as bone, adipose tissue, cartilague and endothelium. They also have a high proliferation rate along with anti-inflammatory and antibacterial capabilities^21–24^. In the last years, different studies have shown that oral tissues, as gingival mucosa, offer a promising source of MSCs for regenerative therapies. More specifically, human gingival mesenchymal stem cells (hGMSCs) have been extensively studied recently as they are relatively easy to isolate from the oral mucosa^25,26^. Compared to other cell sources, hGMSCs show both a higher proliferation rate and higher anti-inflammatory and antibacterial capabilities^27,28^.

The combination of CaP bioceramics with MSCs has demonstrated to promote both proliferation and differentiation of stem cells, providing functional osteoblasts capable of forming new bone^29^. While there is evidence for the potential use of hGMSCs in combination with CaP bioceramics for bone regeneration^30^, its clinical usage is not yet widespread. This is mainly because there is still research to be conducted to fully understand how these cells interact with bioceramics along time to eventually heal a wound or restore bone in a controlled regenerative therapy. Therefore, new investigations focused on the interface between biomaterials and cells are essential for regenerative therapies.

It is known that for the process of osteogenic regeneration to occur, different stages are required. In this sense, cellular activities that are most influenced by bioceramic properties are adhesion, spreading, migration, proliferation and differentiation, and for all these activities cellular communication is essential^31^. Moreover, it is well established that a crosstalk at the cell–material interface occurs from the very beginning of the cell-biomaterial interaction^32,33^, and this crosstalk has a profound influence on cell behaviour, leading in some cases to a cellular network that results in the so-called cellular bridge. This complex cellular structure is considered as the prior step of cellular differentiation process and provides a strong evidence for cell-biomaterial biocompatibility. Furthermore, the biocompatibility process between cells and CaP bioceramic interaction through cellular bridge formation has been scarcely explored. Therefore, further developments in the cellular networks will facilitate closer insights and more profound understandings at the cell–biomaterial interaction levels. Hence, this process is crucial for a regeneration therapy to be succesfull and the product resulting from the CaP bioceramic and hGMSCs interaction deserves a deeper analysis.

According to previous studies combining CaP bioceramics and MSCs, one of the major features allowing for the repair and mineralization of damaged tissue is the bioceramic microarquitecture such as: topography, porosity and pore interconnectivity. Moreover, the role of local geometry and specially that of surface curvature, also has an influence on cell behaviour, although according to our best knowledge there is still a lack in agreement in the field^34,35^. All these bioceramic parameters are known to play a crucial role in cell growth and adhesion, nutrient exchange, cellular proliferation and the formation of a fibrose structured net, prior stage for the formation of cellular differentiation^36^. Hence, due to the importance of morphological and geometrical properties of CaP bioceramics on osteogenic differentiation^8,29^ further studies are required to develop a more cost effective osteogenic strategies to promote bone regeneration^37,38^. So, to proceed in clinical applications integrating both CaP bioceramics and hGMSCs, a more comprehensive knowledge relating the microarchitecture of the bioceramic with the efficiency of the formation of an optimal cellular structure is required.

In this study, cellular networks derived from the hGMSCs-CaP bioceramic interaction were analyzed. Biological performance of CaP bioceramic with macro and microporosity and hGMSCs interaction was further evaluated through cell adhesion and distribution, cellular proliferation and potential osteogenic differentiation.

The purpose of this study was to investigate in vitro cellular networks derived from human gingival mesenchymal stem cells (hGMSCs) and calcium phosphate (CaP) bioceramic interaction.

## Results

### hGMSCs characterization

Cells derived from human gingival tissue were cultured up to the third passage and used for characterization experiments. The immunophenotype was determined by flow cytometry and confirmed for some markers by immunofluorescence (IF). Cells were 100% positive for CD44, CD90, CD73 and CD105 and completely negative for leukocyte markers CD45 and CD34 (Supplementary Fig. 1a) according to the criteria proposed by the Mesenchymal and Tissue Stem Cell Committee of the International Society for Cellular Therapy^39^, (Supplementary Fig. 1A–G). Moreover, hGMSCs displayed osteogenic differentiaton capacities under specifics inductions evidenced through a mineralized matrix determined by Alizarin Red S staining (Supplementary Fig. 1J). Our results are consistent with previous flow cytometry analysis^28^.

**Figure 1:**
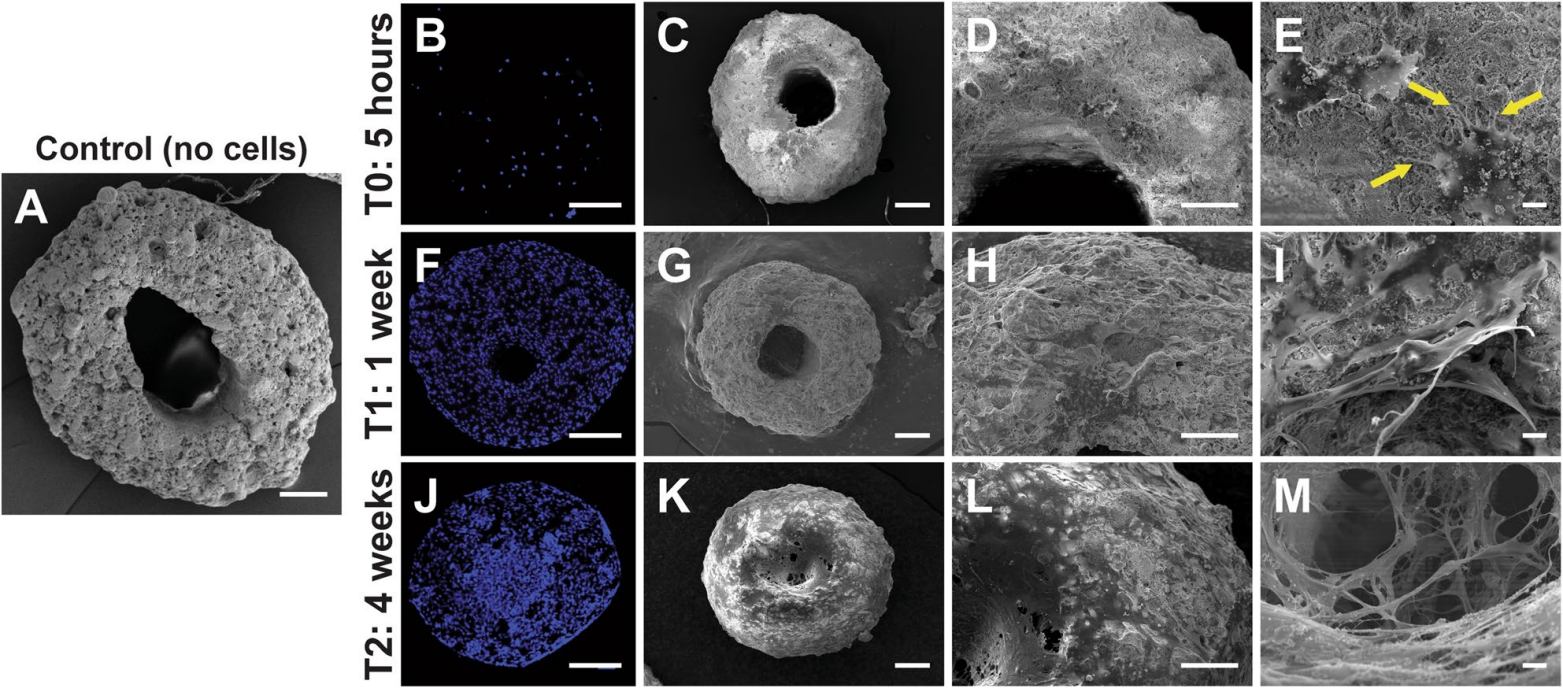
hGMSCs optimally adhere and interconnect when seeded on a CaP bioceramic. Morphology, adhesion and distribution in a CaP bioceramic in three time periods showing cell elongation and total covering of the bioceramic after 4 weeks in culture. (**A**) Control: Representative SEM (scanning electron microscopy) image showing control condition (CaP bioceramic without hGMSCs). Scale bar is 200 μm. (**B,F,J**) Representative fluorescent Confocal microscopy image showing a CaP bioceramic seeded with hGMSCs after 5 h, 1 week and 4 weeks in culture (T0, T1 and T2 respectively). Images represent maximum projections from Z-Stack reconstructions obtained with Confocal Microscopy. Cell distribution is analyzed using Hoechst nuclear staining (shown in blue). Scale bar is 200 μm. (**C,D,E**) Representative image obtained with SEM showing cellular morphology and adhesion of hGMSCs seeded on a CaP bioceramic after 5 h in culture (T0). Cells present a flattened morphology with cell filopodia extending to increase the contact with other cells (E, arrows). Scale bar is 200 μm (**C**), 100 μm (**D**) and 10 μm (**E**), respectively. (**G,H,I**) Representative image obtained with SEM showing cellular morphology and adhesion of hGMSCs seeded on a CaP bioceramic after 1 week in culture (T1). Cells at this period cover different CaP bioceramic areas (**H**), showing an elongated morphology, with extending projections and an initial connection pattern with adjacent cells (**I**). Scale bar is 200 μm (**G**), 100 μm (**H**) and 10 μm (**I**), respectively. (**K,L,M**) Representative image obtained with SEM showing cellular morphology and adhesion of hGMSCs seeded on a CaP bioceramic after 4 weeks in culture (T2). Note how cells cover the whole macropore of the CaP bioceramic at this time period (L, middle area). Higher magnification of this cellular layer reveals an interconnected cellular network with different depths (**M**). 200 μm (**K**), 100 μm (**L**) and 10 μm (**M**). (**E**, **L** and **M**) are higher magnifications of squared areas in **D**,**H** and **L**.

### hGMSCs morphology, adhesion and distribution in the CaP Bioceramic

Previous findings are consistent with MSCs properties described by the literature, indicating that single colony derived hGMSCs represent a putative MSC population with clonogenic renewal and osteogenic differentiation capacities. The initial culture showed a fibroblast-like spindle shape that displayed strong adherence to the base of the plate and after 1 week in culture cells showed a fusiform and elongated morphology. A similar morphology was observed after 1 week in culture in the bioceramic, concluding that hGMSCs maintain their fusiform shape in the presence of the bioceramic (Supplementary Fig. 1h). Cells tend to connect to other cells communicating and sensing the environment in which they are placed, showing the ability to recognize and interact with that milieu^40^. Therefore, when seeded on bioceramics, cells are closely attached to neighboring cells, forming a cellular network on the bioceramic surface, presenting a flattened morphology with cell filopodia extending to increase the contact with other cells (Fig. 1E, arrows). These flattened cells adhere to the underlying substrate, increasing the cell–material interface area, favouring tissue formation and growth^36^ (Fig. 1L,M). In this scenario, cell attachment depends on the bioceramic´s surface and on its geometrical properties and composition^1^, aspects that may influence cellular metabolism and ionic exchange critical for migration and proliferation, but before that happens adhesion to the substrate needs to be strong and stable^36,41^. The CaP bioceramic used in this study presents geometrical features combining macro and micropores, with different pore size, distribution and clustering, as previously described by^37^ (Fig. 1A). In this context, cellular adhesion and distribution were analyzed in three different time periods: 5 h, 1 week and 4 weeks in culture. After 5 h’ cells presented a uniform distribution in the CaP bioceramic, as shown by nuclear Hoechst staining (Fig. 1B), as well as an initial adhesion to the CaP surface, showing an elongated shape with cleary observable filipodia and lamelipodia structures, which may help cells to attach to the bioceramic surface (Fig. 1E, arrows). As other authors have found in mesenchymal stem cells obtained from gingiva^42,43^, our results show that filopodia make contact with other cells on the bioceramic CaP substrate. After 1 week in culture, cells were homogenously distributed along the CaP surface (Fig. 1F). Moreover, hGMSCs penetrated into the micropores and spreading and connecting to other cells, while cells increasing in number and in cytoplasm membrane projections (Fig. 1H,I). After 4 weeks in culture, cellular distribution observed by nuclear staining is less homogeneous as compared to previous time windows, with an increased cell concentration located in the macropore (Fig. 1J,K). Higher magnification revealed a dense cellular network with different layers where cells are noy easily isolated but heavily interconnected, colonizing micropores and covering the CaP in all depths (Fig. 1L,M), covering all the CaP surface, both the macro and the micropores, leading to the next stage related to cellular proliferation.

### hGMSCs distribution and proliferation quantification in the CaP bioceramic

**Figure 2:**
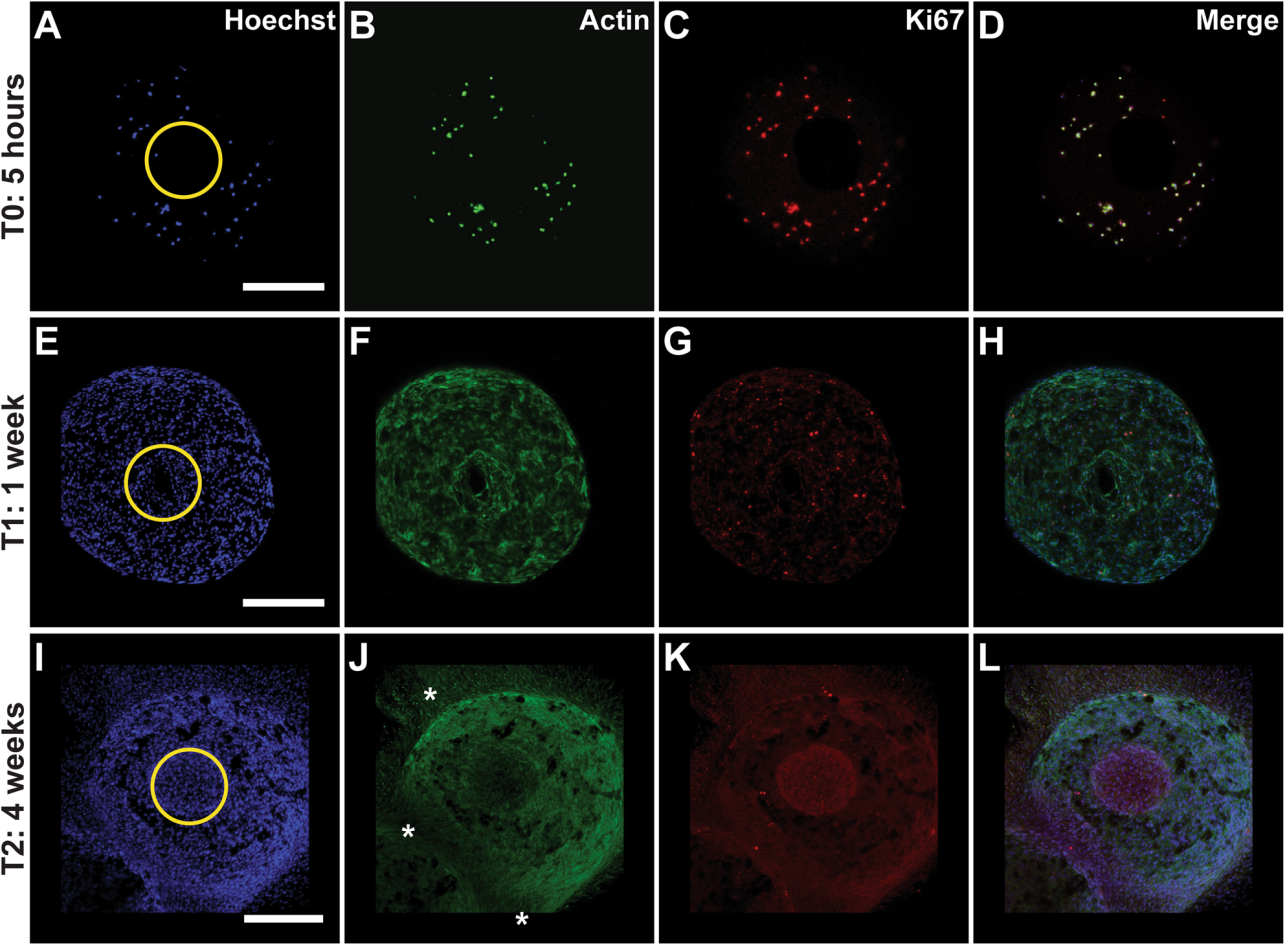
hGMSC present highest proliferation rates after 1 week in culture with a dense cellular network distribution in the CaP macropore after 4 weeks. (**A,B,C,D**) Representative fluorescent Confocal microscopy image showing a CaP bioceramic seeded with hGMSCs after 5 h in culture (T0). In blue, Hoechst nuclear staining (**A**), in green actin (**B**) and red Ki67 proliferation marker (**C**). Merge of three channels is shown in D. Images represent maximum projections from Z-Stack reconstructions obtained with Confocal Microscopy. Scale bar is 200 μm. (**E,F,G,H**) Representative fluorescent Confocal microscopy image showing a CaP bioceramic seeded with MSCs after 1 week in culture (T1). In blue, Hoechst nuclear staining (**E**), in green actin (**F**) and red Ki67 proliferation marker (**G**). Merge of 3 channels is shown in H. Images represent maximum projections from Z-Stack reconstructions obtained with Confocal Microscopy. Scale bar is 200 μm. (**I,J,K,L**) Representative fluorescent Confocal microscopy image showing a CaP bioceramic seeded with hGMSCs after 4 weeks in culture (T2). In blue, Hoechst nuclear staining (**I**), in green actin (**J**) and red Ki67 proliferation marker (**K**). A dense cellular network is observed at this time, with cells spreading outside the CaP bioceramic (**J**, stars). This cellular network will lead to cellular bridges. Note how the macropore area of the CaP bioceramic (yellow circles) becomes completely covered by cells after 4 weeks in culture. Merge of 3 channels is shown in L. Images represent maximum projections from Z-Stack reconstructions obtained with Confocal Microscopy. Scale bar is 200 μm.

The ability of cells to optimally adhere and interconnect when seeded on a CaP bioceramic will have an impact on different cellular stages such as proliferation, known to preceed differentiation (bone formation). Cellular proliferation is related to biocompatibility with the substrate and shows a restricted temporal window, as demonstrated by previous studies^17^. As mentioned before, bioceramics need to reproduce the pore size and pore interconnectivity of real human tissues in order to achieve an optimal cell proliferation, since these parameters are critical for cellular contact, nutrient distribution and waste removal of cells^44^. The proliferation rate of hGMSCs seeded on CaP bioceramics was studied by performing immunofluorescence using the Ab Ki67. In this context, cellular distribution and proliferation were quantified in three different time periods; 5 h, 1 week and 4 weeks in culture and without CaP bioceramic (only cells, data not shown). After 5 h, cells presented a uniform distribution in the CaP bioceramic, as shown by nuclear Hoechst and Actin staining (Fig. 2A,B), with a clear presence of Ki67 in the cell nuclei (Fig. 2C), colocalizing with the nuclear staining Hoechst (Fig. 2D), confirming the specificity of both markers in hGMSCs (Fig. 2H,L).

**Figure 3:**
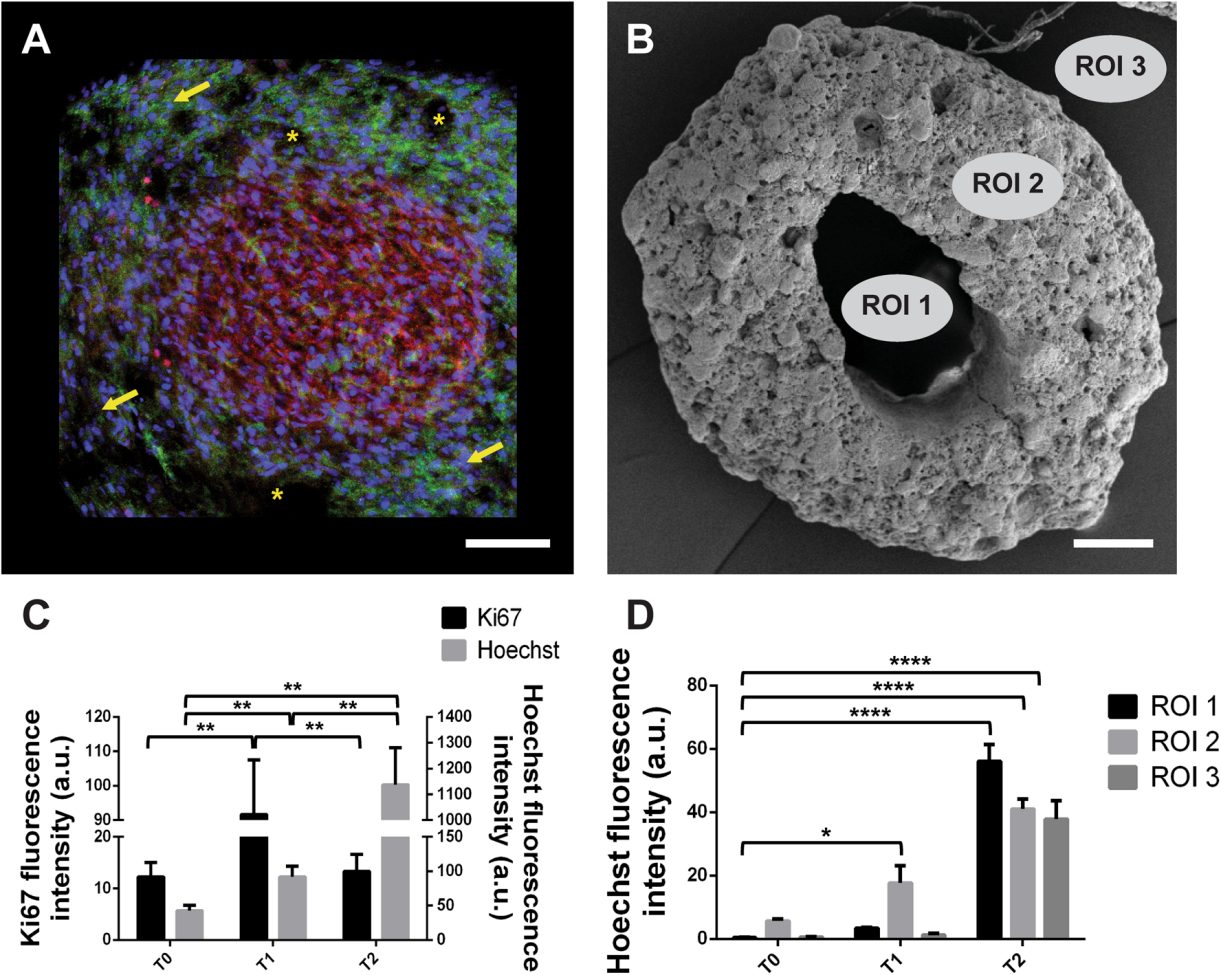
Quantitative analysis obtained by ImageJ software of hGMSCs proliferation along the three time periods shown in Fig. 2. (**A**) Representative image showing a higher magnification of the CaP macropore, indicating hGMSCs and CaP interaction after 4 weeks in culture. Note yellow stars showing empty areas of the CaP and yellow arrows indicating cell nuclei. Note a higher cellular density at the macropore, with a higher expression of Ki67, indicating a proliferative stage of hGMSCs, with cells in the surroundings expressing low levels of this marker. (**B**) Representative image of a CaP bioceramic without hGMSCs, to illustrate the different areas selected for cellular quantification analysis, depicted in (**D**). ROIs correspond to regions of interest selected for quantification: ROI1 corresponds to the macropore area in the CaP bioceramic, ROI 2 is the area in the CaP bioceramic and ROI 3 is the area outside the CaP bioceramic. Quantifications were obtained by ImageJ software. Scale bar is 200 μm. (**C**) Quantitative analysis obtained by ImageJ software of hGMSCs proliferation along the three time periods shown in the panels in Fig. 2 (**A**,**E**,**I** for Hoechst quantification and **C**,**G**,**K** for Ki67). Note highest Ki67 expression levels after 1 week in culture. Hoechst nuclear marker shows highest cell amounts after 4 weeks in culture together with a decrease in cellular proliferation (Ki67). N = 3. Statistical significance *P < 0.05 and ***P < 0.001, verified by One-way ANOVA. (**D**) Quantitative analysis of hGMSC density and distribution in the CaP bioceramic in the three time periods (5 h, 1 week and 4 weeks) obtained by ImageJ software. Note highest fluorescence intensity levels at the macropore (ROI 1) after 4 weeks in culture. N = 3. Statistical significance *P < 0.05 and ***P < 0.001 verified by two-way ANOVA.

After 1 week in culture, cells were homogenously distributed in the CaP surface as shown by nuclear Hoechst and Actin staining (Fig. 2E,F). We further quantified the number of cells that were in active phases of the cell cycle (Figs. 2C,G,K, 3C), depicting highest levels of Ki67 expression observed after 1 week in culture, consistent with previous studies^17,29^. Moreover, an increase in cell density along time is observed, quantified by counting cell nuclei stained by Hoechst (Figs. 2,3D) Statistical analysis using one-way ANOVA revaled highly significant differences among all time periods in terms of cellular number (*P* < 0.001). Tukey’s Honestly Significant Difference (HSD) post hoc test revealed significant differences between pairs; T0–T1 (*P* < 0.05), T0–T2 (*P* < 0.001) and T1–T2 (*P* < 0.05).

Statistical analysis using one-way ANOVA revaled highly significant differences between time periods in Ki67 expression levels (*P* < 0.001). Tukey’s Honestly Significant Difference (HSD) post hoc test revealed highly significant differences between T0–T1 and T1–T2, respectively (*P* < 0.001). Comparison between T0 and T2 showed no significant differences, suggesting that proliferation levels reach a peak by T1, with cells leaving the proliferative stage and start expressing osteogenic differentiation genes by T2. After 4 weeks in culture, cellular distribution observed by nuclear staining is less homogeneous, as compared to previous time windows, with a big cell concentration located in the macropore (Figs. 2I, 3A). Fluorescence intensity quantifications comparing different regions of interest (ROIs) in the CaP at different time points, revealed an increase in fluorescence intensity along time inside the CaP bioceramic with the highest fluorescence intensity signal observed at the CaP macropore after 4 weeks in culture (Fig. 3A,D), followed by lower intensity areas in the surrounding bioceramic and outside it. Statistical analysis using two-way ANOVA (comparing ROIS and time periods) revealed significant differences in the interaction between factors (*P* < 0.05). Comparison between different time periods (T0, T1 and T2) also revealed highly statistical differences (*P* < 0.001) as well as comparison between different ROIs (1, 2 and 3) (*P* < 0.05). Tukey’s Honestly Significant Difference (HSD) post hoc test showed statistical differences between pairs at different times and ROIs, specifically between T0-ROI1 and T1-ROI2, between T0-ROI1 and T2-ROI1, between T0-ROI1 and T2-ROI1 and between T0-ROI1 and T2-ROI3. It is worth mentioning that deeper observations in the area outside the CaP (Fig. 3B, ROI 3) revealed a dense cellular network, with cells spreading outside the CaP bioceramic (Fig. 2J, stars), and forming the so-called cellular bridges, that will be described in the following section.

Together, these results argue strongly that the analyzed CaP bioceramic is not only biocompatible but also it promotes cellular proliferation, which lead us to examine the potential osteogenic differentiation of this cell population.

### CaP bioceramic and GMSCs interface: cellular bridges quantification

Physical contact between cells is a direct form of cellular communication that has been shown to result in the formation of cellular bridges, created through cytoplasmic channels and allowing the transferring of signals and molecular components between cells. These bridges allow a direct interaction between cells in order to ensure the different cellular stages such as proliferation and differentiation^31^.

**Figure 4:**
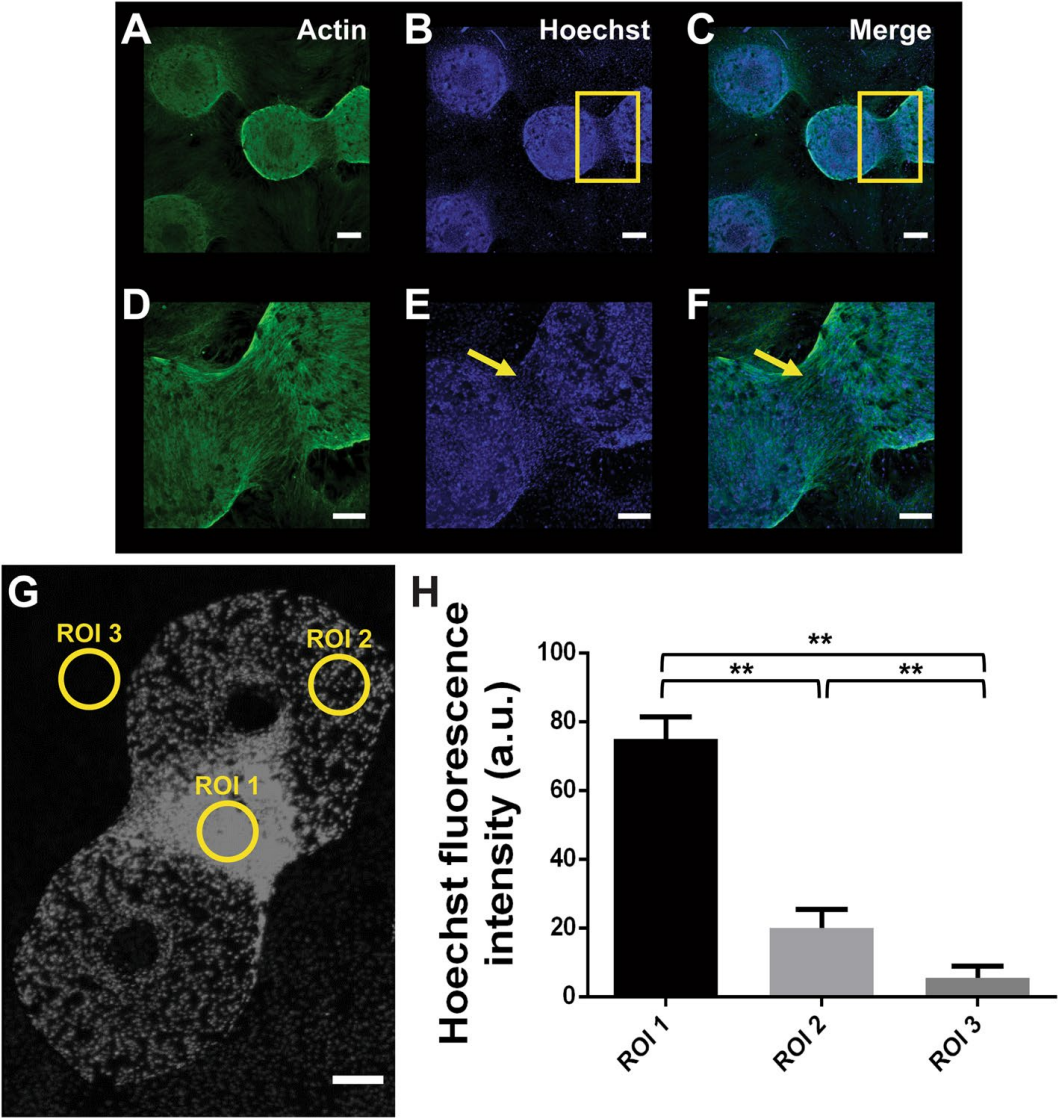
CaP Bioceramic and hGMSCs interface leads to cellular bridge formation after 4 weeks in culture. (**A,B,C**) Representative fluorescent Confocal microscopy image showing two CaP bioceramics seeded with GMSCs after 4 weeks in culture. In green actin (**A**), in blue Hoechst nuclear staining (**B**), and in C merge of both channels. Images represent maximum projections from Z-Stack reconstructions obtained with Confocal Microscopy. Scale bar is 200 μm. (**D,E,F**) Higher magnification from the squared areas in a-c focusing on the bridges that arise between CaP bioceramics, connecting them and showing an elongated cell morphology. Images represent maximum projections from Z-Stack reconstructions obtained with Confocal Microscopy. Scale bar is 200 μm. (**G**) Representative image of two CaP bioceramics connected by a cellular bridge after 4 weeks. Different regions of interest (ROIs) in the CaP bioceramic were selected to illustrate how cellular quantification in different areas was performed: ROI 1 is the area connecting the two CaP bioceramics (cellular network or bridge), ROI 2 is the area in the CaP bioceramic and ROI 3 is the area outside the bioceramic. Scale bar is 200 μm. (**H**) Graph depicting quantification of cellular fluorescence intensity in two CaP bioceramics seeded with hGMSCs and quantified after 4 weeks (T2) in culture obtained by ImageJ software. Highest fluorescence intensity was observed in the cellular bridge connecting the two bioceramics (ROI 1) as compared to other areas either in the bioceramic or in the area outside the bioceramic (ROI 2 and 3 respectively). N = 6. Statistical significance *P < 0.05 and ***P < 0.001, verified by One-way ANOVA.

The quantification of cellular bridges was performed after 4 weeks in culture using actin as cytoplasmatic marker and Hoechst for nuclear staining. We observe a cellular matrix connection between CaP bioceramics and the plate and also between CaP bioceramics in some cases (Fig. 4A). Higher magnification of these bridges revealed the formation of a new complex macrostructure arising from cell–cell contact (Fig. 4D) and probably also from migrating cells coming from the CaP bioceramic, consistent with the fact that no cells were observed at these locations at earlier stages (data not shown) and also with observation of cell nuclei in the bridges (Fig. 4E,F). Quantification of fluorescence intensity levels of cellular nuclei observed at three different CaP locations (Fig. 4G,H) revealed a higher cell density in cellular bridges between CaP bioceramics (ROI1). Statistical analysis among groups using 1-way ANOVA revaled highly significant differences between all ROIs (*P* < 0.001). Tukey’s Honestly Significant Difference (HSD) post hoc test revealed highly significant differences between pairs, namely between ROI1–ROI2 and ROI1–ROI3 (*P* < 0.001); and ROI2-ROI3 (*P* < 0.05).

In line with our previous quantifications of Ki67 expression (Fig. 3C), we can argue that this cell population may correspond to cells that already exit the proliferative stage and that might be in the state to express osteogenic differentiation genes.

### Cellular bridges derive from CaP bioceramic hGMSCs

**Figure 5:**
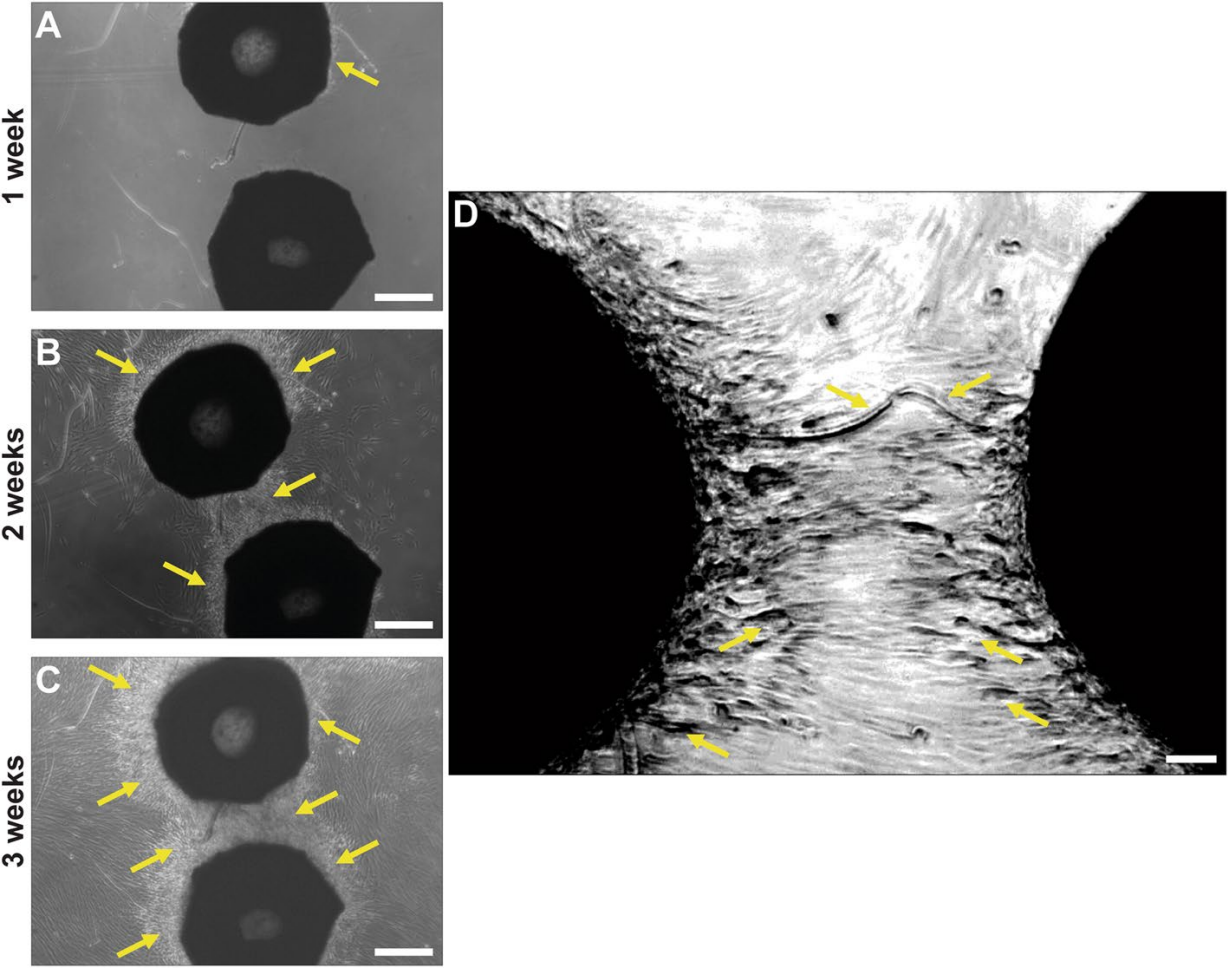
hGMSCs forming cellular bridges might have their origin in the CaP bioceramic. (**A**) Representative optical microscopy image showing CaP bioceramics seeded with hGMSCs. This bioceramic-cells complex comes from a 1 week culture that was trespassed to a new plate. Note hGMSCs absence in the plate. Scale bar is 200 μm. (**B**) Evolution of CaP bioceramics in (**A**) after 2 weeks in culture. Note increased cell density around bioceramics and between them (arrows). Scale bar is 200 μm. (**C**) Evolution of CaP bioceramics in (**A**) after 3 weeks in culture. Note increased cell density around the bioceramics and between them, already showing a cellular bridge (arrows). Scale bar is 200 μm. (**D**) Representative higher magnification after 3 weeks in culture, focusing on the connecting cellular projections from two different CaP bioceramics arising at different areas (arrows). Note cellular elongations connecting and forming cellular networks.

In order to provide evidence that cells observed in the bridges derive from the CaP bioceramics and not from the plate where hGMSCs are seeded on the bioceramics, the CaP bioceramics were trespassed to a different plate after 1 week of culture (Fig. 5A), observing an increase in cell density (Fig. 5B) as well as the formation of a cellular bridge after 2 weeks in culture in the new plate (Fig. 5C). This confirms that cells forming the new and independent macrostructure known as cellular bridge have their origin in the CaP bioceramic and suggests a dynamic cell–cell contact mechanism on which cells extend their projections in order to create a cellular network that preceeds osteogenic formation (Fig. 5D).

### CaP bioceramic induces the expression of preosteoblasts marker OPN in vitro

Cellular differentiation is a crucial stage for the development of an osteogenic matrix that will result in the formation of new bone in combination with the CaP bioceramic. In this sense it was important to analyze whether the presence of the CaP bioceramic was enough for hGMSCs to upregulate the expression of a specific osteogenic differentiation. To do so, we used the specific marker osteopontin (OPN), a protein produced by mature osteoblasts in the process of bone formation and known to be a key marker of osteogenic differentiation, which expression increases in differentiation processes^4,45–47^. We evaluated the in vitro expression of OPN both at the mRNA and protein levels. In this context, OPN expression was analyzed in three different time windows; 5 h, 1 week and 4 weeks in culture in the absence of osteogenic induction compounds and compared to the control condition (only cells).

**Figure 6:**
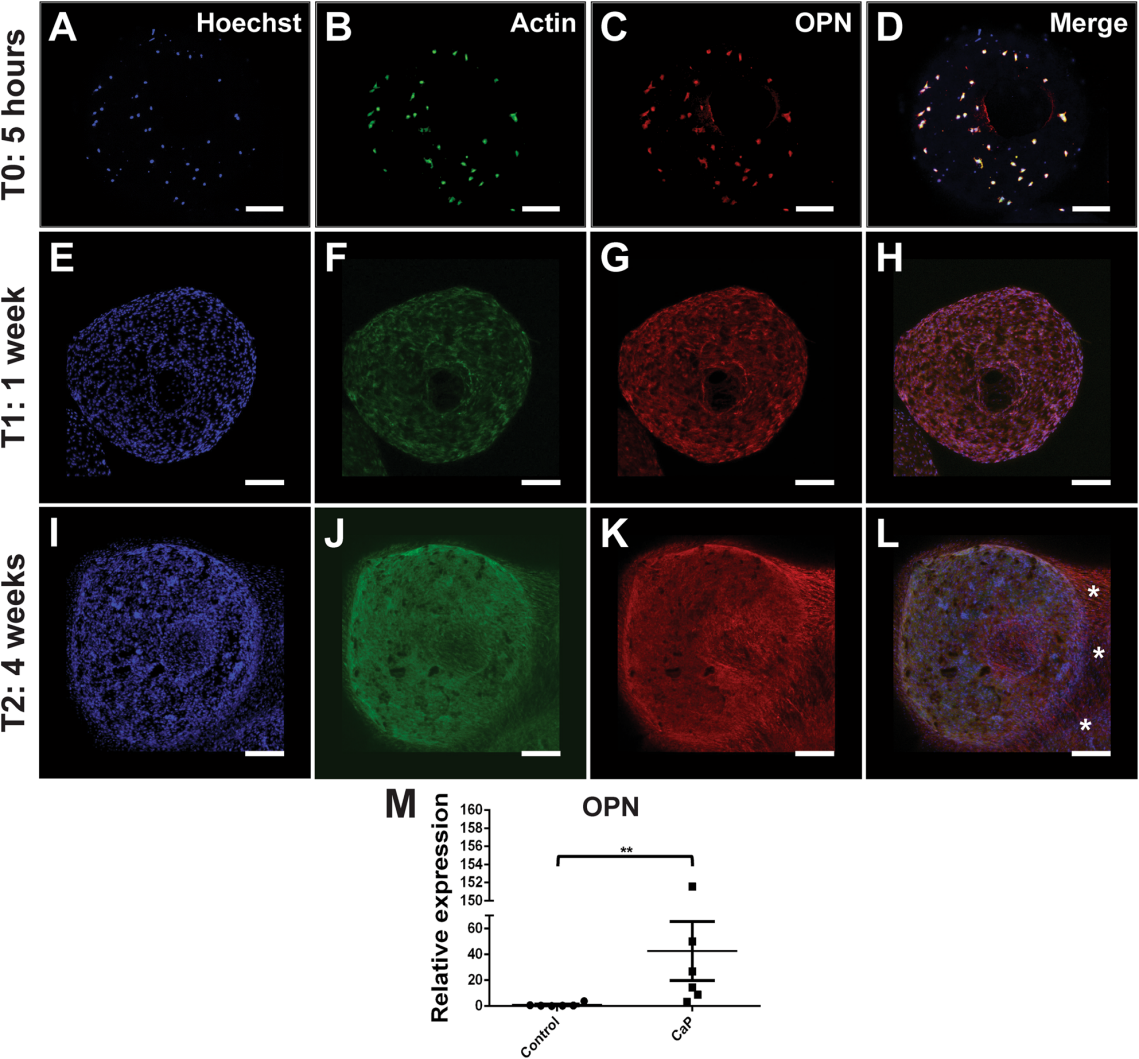
Osteoinductible effect of the CaP bioceramic on hGMSCs in culture. (**A,B,C,D**) Representative fluorescent Confocal microscopy image showing a CaP bioceramic seeded with hGMSCs after 5 h in culture (T0) in the absence of osteogenic induction. In blue, Hoechst nuclear staining (**A**), in green actin (**B**) and red OPN (**C**). Merge of 3 channels is shown in (**D**). Images represent maximum projections from Z-Stack reconstructions obtained with Confocal Microscopy. Scale bar is 200 μm. (**E,F,G,H**) Representative fluorescent Confocal microscopy image showing a CaP bioceramic seeded with GMSCs after 1 week in culture (T1) in the absence of osteogenic induction. In blue, Hoechst nuclear staining (**E**), in green actin (**F**) and red OPN (**G**). Merge of 3 channels is shown in (**H**). Images represent maximum projections from Z-Stack reconstructions obtained with Confocal Microscopy. Scale bar is 200 μm. (**I,J,K,L**) Representative fluorescent Confocal microscopy image showing a CaP bioceramic seeded with hGMSCs showing an increase in OPN expression after 4 weeks in culture (T2) in the absence of osteogenic induction. In blue, Hoechst nuclear staining (**I**), in green actin (**J**) and red OPN (**K**). Merge of 3 channels is shown in (**L**). Images represent maximum projections from Z-Stack reconstructions obtained with Confocal Microscopy. Note the dense cellular network at this time, with cells spreading outside the CaP bioceramic (**L**, stars). Scale bar is 200 μm. (**M**) Graph depicts relative expression of OPN in hGMSCs seeded with CaP bioceramics at day 21 obtained by qPCR, showing an increase in mRNA expression in OPN as compared to the control condition, differences were statistically significant *P < 0.05 verified by Mann Whitney U test, Non-parametric test N = 3 biological replicates with two technical replicates each.

Our results showed that, consistent with previous literature, OPN expression increased its expression along time, consistent with a higher cellular density previously described (Fig. 3D)^48^. Moreover, OPN expression, from a qualitative perspective, increased along time both at the micro and macropore levels (Fig. 6C,G,K) as well as at the bridges (Fig. 6J,K,L, stars) is observed. Additionally, we performed qRT-PCR after 3 weeks in culture measuring OPN mRNA expression levels. Our results showed an increase in mRNA expression in OPN as compared to the control condition (Fig. 6, graph M). Statistical analysis using Mann–Whitney U test to compare two independent groups revealed significant differences in OPN expression between control condition and CaP bioceramic with hGMSCs by day 21 (*P* < 0.05). To conclude, our analysis revealed that OPN was upregulated during cell culturing, demonstrating an osteoinductible effect of the CaP bioceramic on hGMSCs.

## Discussion

The rapid increase in ageing worldwide will soon lead to different health requirements, being osteogenic regenerative therapies one of the main challenges to be approached in the clinic. In the current study, a CaP bioceramic with both micro and macropores in combination with hGMSCs was used in order to analyze their interaction and biocompatibility process along time in vitro for potential clinical applications. For this purpose, morphological analysis, cellular and molecular biology techniques were used. Moreover, adhesion, distribution, proliferation and osteogenic potential differentiation of hGMSCs were investigated, and cellular networks derived from cellular connectivity and CaP bioceramic-hGMSCs interaction were described, enriching a relatively little explored concept and providing new insigths into the field.

The relative novel cellular population used in this research (hGMSCs) whose isolation method has been carried out by different authors^49–51^ constitutes an optimal cell source for therapy since it allows in vitro studies^30^, and expresses all the literature described markers required for its characterization (Supplementary Fig. 1). Moreover, hGMSCs as shown in the literature^51,52^ have higher anti-inflammatory and proliferative levels than other stem cell sources such as bone marrow, which can potentiate the basic processes that precede the formation of mayor complexity cellular structures, according to the substrate where they are seeded. hGMSCs also show contact inhibition when they reach confluency in culture (data not shown), but certain substrates may allow a sustained proliferation stage until a new differentiation process occurs^28,30^. On the other hand, CaP bioceramic used in the current study has been considered ideal substrates that efficiently allowed for cellular adhesion and proliferation, as demonstrated previously^17,19,33^. In the current study high proliferation levels were observed until the third week in culture (Fig. 3C), which is mainly achieved due to the CaP composition and geometry, combining micro and macropores, as previously described in^37^. The competent cellular adhesion to the CaP bioceramic was confirmed by the elongated fusiform cellular morphology shown by the SEM panels (Fig. 1), with lamellipodia formation undoubtedly helping to improve cellular linkage to the substrate. Cellular adhesion is the first relevant step for the optimization of a complex cell-substrate interface required to repair and mineralize damaged tissue. In this crucial step, different aspects of the CaP morphology and microarquitecture such as topography, porosity and pore interconnectivity will influence cell attachment, interconnectivity, cellular communication, nutrient exchange and cell proliferation^1,8,29^. For clinical aspects, it is of high importance that the CaP bioceramic provides an adequate cellular niche where cells can embrace-conquer all the possible regions in the effective CaP bioceramic surface, not only adhering to the bioceramic but also proliferating there. Consistent with the literature, the highest proliferation levels in our study were observed after 1 week in culture^17,29^ (Fig. 3C), and the CaP macropore was observed to be filled with cells after 4 weeks in culture (Fig. 2I–L). This CaP cellular filling requires a high cellular adhesion as well as proximity, communication and cellular extension, in order for hGMSCs to develop a cellular network on the bioceramic surface. The presence of this cellular network at early stages of the hGSMCs-CaP interface is a highly relevant step for the cellular communication process and for the formation of macrostructures preceding osteogenic differentiation^31,36^. This cellular network, known as cellular bridge, derives from the hGMSCs–CaP crosstalk may have a profound influence on both cell behaviour and communication, influencing ionic exchange as well as hGMSCs metabolism and activity. Our results show that cellular bridges connect CaP bioceramics (Fig. 4) and arise as a result of the physical contact between cells. Interestingly, hGMSCs forming these bridges were not derived from the seeding process (cells sticking to the plate) but had their origin in the CaP bioceramic itself (Fig. 5). These cellular bridges could allow multiple cell bodies to act synchronously exchanging vesicles, cell-surface components and calcium fluxes and highly depend on the CaP chemical composition^36^. Moreover, they can allow cells to connect and transport different types of messages required for cellular processes, regardless of the distance between them^36^. Cellular bridges clearly depend on the direct interaction between cells, ensuring the different cellular stages such as migration, proliferation and differentiation^31^, preceeding osteoblast differentiation and providing a strong evidence for cell-biomaterial biocompatibility^36^. In line with this, the number of viable cells increased with incubation period, as revealed by statistical analysis; time effects on biomaterial cell interaction can also be analyzed by the current model (Fig. 3C).

As previously reported, hGMSCs constitute an interesting model to study tissue regeneration^28^. In this sense, we observed an increase in the expression of the specific marker osteopontin (OPN), a protein produced by mature osteoblasts in the process of bone formation and commonly used as a marker of osteogenic differentiation^45,47,53^. Moreover, OPN regulates the interaction between cell–matrix and OPN receptors have been described in hGMSCs^54^. Hence, upregulation of OPN constitutes a crucial role in osteogenesis and its expression has been broadly used as mesenchymal cell marker to indicate differentiation process to osteogenic lineage^4,46^. Some biomaterials such as CaP bioceramics show the ability to be osteoinductive following mechanisms associated to the phosphate transport^36,55^ and OPN is found to increase when cells were combined with porous scaffolds, as shown by^56^. In our study, qRT-PCR expression analysis showed an increase of OPN related to the control condition after 3 weeks in culture (Fig. 6). Consistent with the literature, OPN plays a crucial role in the regulation of cell–matrix; the higher the cell number, the higher the OPN expression^57^. We observed the expression of OPN in all cells in the CaP distributed both in the bioceramic and in the cellular bridges, consistent with the role of cellular adhesion to the matrix. Together with its role in cellular adhesion, OPN also promotes cellular migration of MSCs^58^, which may be relevant for the formation of cellular bridges resulting from hGMSCs communication and interaction with CaPs in the present study. Although the expression of OPN in mature osteoblasts is recognized as the major marker of osteogenic differentiation, it is necessary in the future to analyze the expression of other relevant markers for the bone formation process, genes such as ALP, RUNX2, BSP and DMP. -1.^17,49,59^.

The novelty of this study is the growth of hGMSC cells in a defined CaP structure, which produces macro-structures through the generation of cell bridges that can increase bone regeneration.

Interestingly, different materials with different geometrical properties will influence the adhesion, proliferation and the pattern distribution of cells on the materials surface, which will in turn lead to different outcomes in terms of cell survival, cellular bridge formation and differentiation. For instance, it has been shown that microporosity affects the process of osteogenesis mainly by the increased specific surface areas which can offer more protein adsorption sites whithin biomaterials. Also, the capillary force generated by this microporosity can improve the attachment of bone-related cells on the biomaterial surface, with these cells penetrating them even if these micropores are smaller^60,61^. Several studies have shown the cellular distribution, proliferation and complex structure formation when MSCs are combined with CaP bioceramics^17,19^, yet few have shown the application of human gingival as a source of MSCs^62^ which opens alternative source for clinical applications.

In summary, our in vitro research shows that, hGMSCs-CaP bioceramics interaction, combining both micro and macropores lead to an ideal cell adhesion and cell proliferation. The current research confirmed the presence of cellular bridges arising from the interface between hGMSCs and CaP bioceramics. This system can be considered as a promising tool for osteogenic differentiation therapies. Additional studies using different biomaterials considering different surface and microarquitectural properties are required to analyze cellular adhesion, proliferation, networking, and cellular bridge formation in order to asess the biocompatibility process in vitro along time. In terms of the clinical relevance of the study, although in the last decade an increasing number of clinical trials have been performed in order to determine the clinical viability of the use of MSC-based bone and dental regenerative therapies, the implementation of MSC in combination with bioceramics still requires technological development to optimize in vivo the MSC viability, homing and differentiation to ensure the feasibility of their transplantation for clinical applications^63^. Nonetheless, the current study suggests that hGMSC associated with CaP bioceramics may provide beneficial effects on bone regeneration in preclinical models and human clinical applications. Therefore, the combination of hGMSC and CaP bioceramics is greatly promising for bone regeneration in dentistry and orthopedic clinical interventions.

## Materials and methods

### Human gingival mesenchymal stem cells (hGMSCs) isolation and culture conditions

A heterogenic HGMSC population derived from gingival tissue were obtained from human participants after approval by The Institutional Review Board of Universidad Adolfo Ibáñez, IRB-UAI, by the Bioethical research committee (approval number 54/2019). We have also complied with all relevant ethical regulation and biosafety standards from the Manual of Biosafety Standards and Associated Risks-Fondecyt-CONICYT 2018 and approval by IRB-UAI for all experiments that involved isolation, expansion, quality control of hMSCs. All experiments associated with cell analysis were performed in strict accordance with relevant guidelines and regulations previously described. The protocol for human cell and tissue collection used in this study was also approved by the IRB-UAI and a written informed consent was obtained from each volunteer. Human tissue samples were collected from clinically healthy gingiva of subjects who had no history of periodontal disease and a relatively healthy periodontium. A small biopsy (5 mm) was obtained from gingiva of patients (30–40 years old) under local anesthesia. Tissue samples were kept at 4 °C in DMEM w/high-glucose and L-glut (Genesee) supplemented with antibiotics. Then, biopsies were washed 3 × in HBSS (Santa Cruz) and minced into 1–3 mm fragments to subsequent digestion with 4 mg/ml type IV Collagenase (Santa Cruz) for 6 h at 37 °C. Next, the dissociated cell suspensión was plated on 100 × 20 mm Petri culture dishes with 10% FBS (Gibco)/ Penicillin–Streptomycin (Genesse) supplemented DMEM and cultured in an incubator at 37ºC with 5% CO2 and 95% humidity. After 72 h the nonadherent cells were removed. Cell culture medium was replaced every 72 h. Cells count and cell viability were performed using the trypan blue exclusion method. This test is based on the principle that intact cell membranes on live cells exclude trypan blue cell entry, while membrane pores of unviable cells allow the dye cell entry and cells have a blue cytoplasm. Briefly, cells were Trypsin/EDTA detached s (0.05% trypsin, 1 mM EDTA in PBS) centrifuged (400 g) and resuspended in PBS. Then 50 µl cell aliquots were mixed with 0.4% trypan blue solution in PBS, gently mixed and a 10 µl of cell mixture added to hemacytometer and cells counted on using light microscope. Cells were kept in culture until 80% confluency when they were transferred to new plates for further cell characterization analysis (adapted from^20^ and^22^).

### hGMSCs immune-phenotyping

#### Cell characterization

Experiments were carried out using cells derived from 7 different tissues (biopsies obtained from patients). All cells were characterized by flow cytometry and Inmunofluorescence (IF) using specific markers. For cell characterization passage 3 was used (500.000 cells for FC and 40.000 for IF experiments). For IF, only cells were used, polylysine coated glass covers until cells reached confluency. The immunophenotype was determined by flow cytometry and confirmed for some markers by immunofluorescence (IF). Cells were 100% positive for CD44, CD90, CD73 and CD105 and completely negative for leukocyte markers CD45 and CD34. For flow cytometry, PE- or FITC-conjugated mouse mAbs anti-human CD34, CD45, CD73, CD90 and CD105 (AbCam) were used in a Beckman Coulter flow cytometer and FACScan program. For immunofluorescence cell characterization, cells were fixed in 4% paraformaldehyde (Electron) for 20 min after which they were washed five times during 10 min with PBS-T (PBS 1X (Santa Cruz) mixed with 0.1% Triton 100× Sigma-Aldrich) and then incubated for 20 min at room temperature with a blocking solution containing 1% BSA (Themofisher), PBS-T and 10% horse serum (Themofisher),. Blocking solution was removed and GMSCs were incubated with the primary antibodies CD44 or CD105 (AbCam) overnight. Primary antibodies were then removed and hGMSCs were washed four times during 10 min each with PBS-T and further incubated with the coupled secondary-coupled Alexa Fluor 488 (Santa Cruz) at a concentration of 1:1000 and 1 mg/ml Hoechst (Sigma Aldrich) for 2 h at room temperature. Finally, samples were kept in fluoromount and store at 4 °C until imaging, performed with a confocal microscope (Nikon C1 Plus). Samples were analyzed and quantified using the software ImageJ Version 1.52s (adapted from^20^ and^22^).

### Osteogenic differentiation

For differentiation experiments hGMSCs in passage 6 were used. We seeded 50.000 GMSCs per well, with standard culture conditions and a cell control duplicate (without differentiation cocktail) and another duplicate with differentiation medium in a 4 well plate (1.9 cm^2^/well). Osteogenesis was induced by a 100× osteoinduction solution containing dexamethasone (Sigma Aldrich) (100 mM), ascorbate-2-phosphate (Sigma Aldrich) (5 mg/ml) and B-Glycerolphosphate (Sigma Aldrich) (1 M) in supplemented DMEM. The cultures were maintained for 4 weeks with the medium changed every three days. After being inducted, samples were fixed in 4% PFA for 10 min after which 2 × washings with PBS. Then, cells monolayes were subjected to mineralization assay. Briefly, Alizarin Red (Santa Cruz) (2%) was added to the plates and left for 15 min at room temperature. Samples were washed 5 × with distilled water and photographed (adapted from^20^ and^22^).

### Calcium phosphate (CaP) based bioceramic

The selected CaP bioceramic granules (BonePlus Eagle Eye MegaGen) present a doughnout like shape and a certain topography and a diameter of max 1 mm. These granules are made of synthetic β-TCP/HA composite (60–40%) and each one presents an interconnected channel structure with an average diameter of 50 mm. These CaP bioceramics have been extensively described and analyzed previously^30^.

### Morphological analysis

2 × 10^4^ hGMSCs were seeded on bioceramics placed in 12 well plates (3.8 cm2/well). 20 mg of CaP bioceramics were placed in each well (≈ 30 particles). At the indicated incubation periods cells were fixed for 20 min in buffered 4% glutaraldehyde (Electron) /0.2 mol/L sodium cacodylate solution at 4ºC. Then, fixed cell-bioceramic samples were processed for Scanning electron microscopy (SEM) imaging to obtain images from the surface of the CaP bioceramic—hGMSCs. Samples were dehydrated in a graded series of alcohols (70%, 80%, 95% and 100% for 20 min each), dried and gold sputtered. Samples were mounted on aluminum stubs with double-sided carbon tape and sputter coated with gold for 30 s at 45 mA (Denton Vacuum Desk V). Subsequently, SEM images were obtained (Jeol JSM IT300LV) (adapted from^20^ and^22^).

### Cellular proliferation and differentiation

hGMSCs in passage 3–4 were used. 4 × 10^4^ GMSCs were used in 12 well Plates, with 30 CaPs in each well and a control condition with no CaPs. Triplicates were used in each condition and three times in culture were analyzed. Culture medium was replaced every 72 h. For SEM imaging (morphological analysis), the same cell amounts were used but the fixation protocol was performed using glutaraldehyde instead of Paraformaldehide. After indicated incubation periods hGMSCs on CaP bioceramics were fixed with 4% para-formaldehyde/PBS and subjected to immunofluorescence analysis for ki67 ab-66155 (Santa Cruz) (expression cell proliferation) or for OPN ab-8448 (Abcam) (osteogenic cell differentiation) and actin sc-8432 (Santa Cruz) for cell cytoskeleton. Briefly, cells were permeabilized 10 min with 0.1% Triton X100 (Santa Cruz)/PBS (Santa Cruz), blocked 1 h with 1% BSA (Santa Cruz), 10% Horse serum (Thermo Fisher), PBS and followed with rabbit anti-OPN antibody (1:500, Abcam) or anti-actin Mab (1:1000. Sigma) overnight incubation at 4 °C. Next, samples were incubated with Alexa Fluor 594 donkey anti-rabbit (AbCam) secondary antibody and 1 mg/ml Hoescht 33,258 nuclear staining (Santa Cruz) in order to dye cell nuclei for cell quantification and also to determine the co-localization with ki67 and OPN. Finally, samples were kept in fluoromount and store at 4ºC until imaging, performed with a confocal microscope (Nikon Eclipse 80i). Two different channel settings were used for the pictures: Alexa Fluor 488 (Santa Cruz) (spectral detector range 405–435 nm) and Alexa 594 (AbCam) (spectral detector range 605–675 nm). One Z-Stack was acquired from each sample (CaP bioceramic with hGMSCs) using the EZ-C1 Nikon software. Samples were scanned every 10 μm. After acquisition, the maximum projection of each stack was obtained. Samples were analyzed and quantified using the software ImageJ (1.52p).

For cellular bridges quantification, circular ROIs (48 × 48 px) were selected using ImageJ software selecting three different areas for analysis; cellular bridge area, bioceramic area and area outside the bioceramic. Mean gray value was measured; 6 ROIs were selected in each bioceramic. A total of 3 bioceramics were used for the analysis in independent experiments.

For proliferation quantification, circular ROIs (70 × 70 px) were selected using ImageJ software selecting three different areas for analysis; macropore area, bioceramic area and area outside the bioceramic. Split channles option was used to separate channels and only quantify the red one (ki67). Mean gray value was measured; 3 ROIs were measured in each bioceramic. A total on 3 bioceramics were used for the analysis.

### Quantitative RT-PCR

hGMSCs in passage 3 were used. We performed the experiments using 4 wells per condition, in a 12 wells plate. 40.000 cells in 200 µl DMEM were used. Incubation was for 1 h in CaP and additional DMEM was added (2 ml). CaP bioceramics seeded with hGMSCs were treated with TRI Reagent (Ambion) for total RNA purification. Subsequently, RNA samples were treated with RQ1 DNAse (Promega), with 1 µg of each sample used for cDNA synthesis, using an ImProm-II Reverse Transcription System with oligo (dT) primers (Promega). qPCRs were done in an Eco Real-Time PCR System (Illumina), using 10 µl reactions with a PowerUp SYBR Green Master Mix (Applied Biosystems). The following primers were used at a concentration of 0.8 µM: OPN 5′TGAGAGCAATGAGCATTCCGATG3′, 3′CAGGGAGTTTCCATGAAGCCAC5′^64^; GADPH 5′TCAGCAATGCCTCCTGCAC3′, 3′TCTGGGTGGCAGTGATGGC5′^65^. GAPDH was used as a reference gene for signal normalization. Each qPCR reaction was performed in three or four biological replicates. Additionally, qPCR reactions with No-RT reaction or nuclease-free water as templates were used as negative controls, to prevent for genomic DNA or qPCR mix contamination, respectively.

### Statistical analysis

In order to investigate hGMSCs distribution and proliferation along the three time periods, Hoechst expression and Ki67 expression were measured. The single factor of CaP bioceramic regions of interest (ROIs) along with the factor of time in culture (T0, T1 and T2), one-way and two-way ANOVA were used, respectively. Also, Tukey’s Honestly Significant Difference (HSD) post hoc test was conducted using the statistics packageTest of GraphPad (Prism 8). These results were expressed as means and standard deviations. To compare OPN expression levels Mann–Whitney U test was performed, results are reported as means and standard errors. In all cases the significance level was set at α = 0.05. All experiments were performed with at least three individual replicates. Assumptions of normality and equality of variance were checked by Shapiro–Wilk and Levene´s test, respectively.

## Supplementary information

Supplementary file 1.

Supplementary file 2.
