# Supplementary material for: Analysis of cell-biomaterial interaction through cellular bridge formation in the interface between hGMSCs and CaP bioceramics
Source: Sci Rep. 2020 Oct 5;10:16493. doi: 10.1038/s41598-020-73428-y (PMC7536240; doi:10.1038/s41598-020-73428-y)
Supplement: Supplementary file 1 [file 41598_2020_73428_MOESM1_ESM.pdf]

Title: Analysis of cell-biomaterial interaction through cellular bridge formation in the interface between hGMSCs and CaP bioceramics.

Authors: Isabel Benjumeda Wijnhoven[2] Raúl Vallejos Baier[1], Juan F. Santibanez[3], Carola Millán Giovanetti[2], Juan F. Vivanco[1,*].

1- Faculty of Engineering and Sciences, Universidad Adolfo Ibáñez, Viña del Mar, Chile
2- Faculty of Liberal Arts, Universidad Adolfo Ibáñez, Viña del Mar, Chile.
3- Group for Molecular Oncology, Institute for Medical Research, University of Belgrade, Serbia.

Keywords: cellular and biomaterial interaction, hGMSCs, cellular network, calcium phophate biomaterials.

* corresponding author: juan.vivanco@uai.cl

Supplementary material

Supplementary Figure 1. Human Gingival Mesenchymal Stem Cells (hGMSCs) present Mesenchymal Stem Cells (MSCs) typical markers and display osteogenic differentiation ability under specific induction.

A. Representative image of cell characterization obtained by Flow cytometry using CD90, 73 and 105 mesenchymal markers showing that 100% of hGMSCs are positive for the markers, (table). On the other hand, 0% of hGMSCs were positive for hematopoietic markers CD45 and 34 (table). N=7.

B-G. hGMSCs characterization by immunofluorescence using MSCs markers. Cells were immunostained with mouse antibodies (Abs) specific for human CD44 (Fig. B, C, D) and CD105 (Fig. E, F, G) followed by incubation with Alexa 488 conjugated secondary Ab. Nuclei were stained using Hoechst (Fig. B, E). Merge of the two channels (Hoechst and CD44 or 105) are depicted in images D and G, respectively. Images represent maximum projections from Z-Stack reconstructions obtained with Confocal microscopy. Scale bar is 100 μm.

H, I, J. Representative image of hGMSCs after 3 weeks in culture showing cells with fusiform morphology and 80-100% confluency (H). Fig. J depicts cell culture after 4 weeks supplemented with osteoinduction solution and stained with Alizarin Red. Note calcium depositions in the darker areas of the image (J). Control: hGMSC in culture without osteoinduction solution and stained with Alizarin Red (I). Scale bar is 200 μm.
