# Supplementary figures and images for: Analysis of cell-biomaterial interaction through cellular bridge formation in the interface between hGMSCs and CaP bioceramics
Source: Sci Rep. 2020 Oct 5;10:16493. doi: 10.1038/s41598-020-73428-y (PMC7536240; doi:10.1038/s41598-020-73428-y)

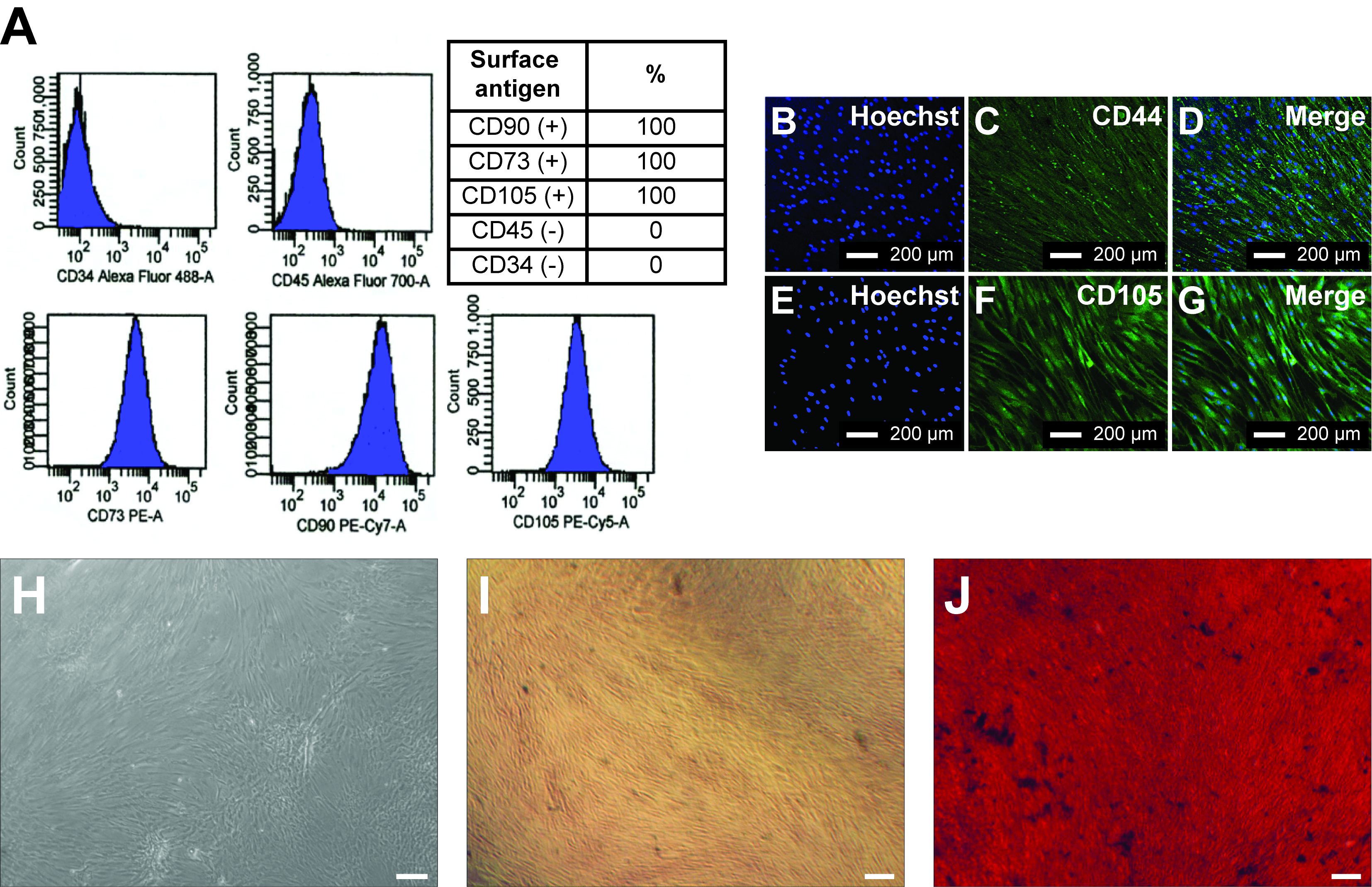

Supplement: Supplementary file 2 [file 41598_2020_73428_MOESM2_ESM.tif]
